# A genogeographic study of the Kyrgyz mountain merino via microsatellite markers

**DOI:** 10.18699/VJGB-23-22

**Published:** 2023-04

**Authors:** A.В. Bekturov, Zh.T. Isakova, V.N. Kipen, T.Dzh. Chortonbaev, S.B. Mukeeva, S.K. Osmonaliev, К.А. Aitbaev

**Affiliations:** Kyrgyz National Agrarian University named after K.I. Skryabin, Bishkek, Kyrgyz Republic; Research Institute of Molecular Biology and Medicine, Bishkek, Kyrgyz Republic; Institute of Genetics and Cytology of the National Academy of Sciences of Belarus, Minsk, Republic of Belarus; Kyrgyz National Agrarian University named after K.I. Skryabin, Bishkek, Kyrgyz Republic; Research Institute of Molecular Biology and Medicine, Bishkek, Kyrgyz Republic; Kyrgyz Research Institute of Animal Husbandry and Pastures, Sokuluk District, Kyrgyz Republic; Research Institute of Molecular Biology and Medicine, Bishkek, Kyrgyz Republic

**Keywords:** Kyrgyz mountain merino, genotyping, STR markers, киргизский горный меринос, генотипирование, STR-маркеры

## Abstract

The aim was to ascertain the genetic and geographical structure of the Kyrgyz mountain merino (KMM). We analyzed DNA samples of 109 Kyrgyz mountain merino specimens, bred in three state breeding factories (STB), including “Orgochor” in the Issykul Province, “Katta-Taldyk” in the Osh Province and STb named after Luschikhin in the Talas Province. We identified 126 alleles in 12 microsatellite markers (McM042, INRA006, McM527, ETH152, CSRD247, OarFCB20, INRA172, INRA063, MAF065, MAF214, INRA005, INRA023). There were 6 to 16 alleles in each locus (mean 10.500 ± 0.957 alleles per locus). We identified 67 rare alleles (prevalence less than 5.0 %), which made up 53.2 % of all alleles found. The greatest number of rare alleles was found in STR-markers of CSRD247, INRA023, INRA005, INRA006, MAF214 and OarFCB20. For each group, there were individual differences in the distribution of allele frequencies across all the STR loci studied. The most significant of them were as follows: with regard to the McM042 locus, allele 87 was major in the TALAS and OSH groups (35.6 and 45.7 %, respectively), whereas allele 95 was major in the ISSYK- KUL group (36.2 %); allele 154 was major in all groups with regard to the INRA172 locus, but it was 1.25 times less prevalent in the ISSYK-KUL and 1.66 times less prevalent in the OSH groups compared to TALAS (55.2 and 41.4 %, respectively), whereas alleles 156 and 158 were found only in the ISSYK-KUL group. Considering the ETH152 locus, 186 allele prevalence in the TALAS group was 51.1 %, but allele 190 was also markedly prevalent in the ISSYK-KUL and OSH groups, 34.5 and 34.3 %, respectively. The genetic division of the studied groups of KMM (with K from 3 to 10) was homogeneous – the contribution of each subcluster was equivalent. The AMOVA analysis revealed that the groups are located equidistantly. To conclude, the genetic diversity of the Kyrgyz mountain merino in three state breeding factories of the Kyrgyz Republic was high and comparable with each other.

## Abstract (ru)

Проведено геногеографическое изучение породы овец киргизский горный меринос (КГМ). Проанализированы образцы ДНК 109 овец данной породы, разводимых в трех государственных племенных заводах (ГПЗ) в Республике Кыргызстан: ГПЗ «Оргочор» (Иссык-Кульская область), ГПЗ «Катта-Талдык» (Ошская область) и ГПЗ им. М.Н. Лущихина (Таласская область). В 12 исследованных микросателлитных маркерах (McM042, INRA006, McM527, ETH152, CSRD247, OarFCB20, INRA172, INRA063, MAF065, MAF214, INRA005, INRA023) идентифицировано 126 аллелей. Число аллелей в каждом локусе варьировало от 6 до 16 при среднем значении 10.500 ± 0.957 аллелей на локус. Определено 67 редких аллелей (с частотой встречаемости менее 5.0 %), что составляет 53.2 % от общего количества выявленных аллелей. Наибольшее количество редких аллелей установлено для STR-маркеров CSRD247, INRA023, INRA005, INRA006, MAF214 и OarFCB20. Для каждой группы имеются индивидуальные различия в профиле распределения частот аллелей по всем исследуемым STR-локусам, наиболее значимые из которых следующие: в группах TALAS и OSH для локуса McM042 в мажорном состоянии находится аллель 87 (35.6 и 45.7 % соответственно), в то время как для группы ISSYK-KUL наибольшую распространенность получил аллель 95 (36.2 %); для локуса INRA172 во всех группах мажорным аллелем был 154, однако в сравнении с группой TALAS его распространенность была меньше в 1.25 (ISSYK-KUL) и 1.66 (OSH) раза – 55.2 и 41.4 % соответственно, а аллели 156 и 158 встречались только в группе ISSYK-KUL; для локуса ETH152 частота встречаемости аллеля 186 в группе TALAS составила 51.1 %, для групп ISSYK-KUL и OSH значительную распространенность приобретает аллель 190 – 34.5 и 34.3 % соответственно. При оценке генетической подразделенности исследуемых выборок КГМ (при К от 3 до 10) показана однородность струк- туры – вклад каждого субкластера равноценный. При анализе AMOVA обнаружено, что выборки расположены равноудаленно. Таким образом, генетическое разнообразие овец породы КГМ среди трех государственных племенных заводов Кыргызской Республики достаточно высокое и сопоставимое между собой.

## Introduction

The sheep breeds of Kyrgyz mountain merino (KMM) are common in all regions of the Kyrgyz Republic, which differ in natural and climatic conditions. In order to improve the breeding and productive qualities of KMM sheep, intra-breed (zonal) types of sheep were created (Bekturov et al., 2017).

The Kyrgyz mountain merino was created in 1990–2006 on the basis of the Kyrgyz fine-wool breed using sheep of the Australian merino breed and approved in 2006. The genetic structure of the breed includes 5 factory types and 24 factory lines. KMM sheep wool has high technological properties and has attributed to the highest quality categories of merino fine wool. The sheep are also known for high meat properties

Each breed or animal type shows some heterogeneity in morphological, productive and technological qualities. Microsatellite loci (short tandem repeat, STR) can be used to solve breeding tasks related to the determination of breed affiliation or breed type (Deniskova et al., 2018; Isakova et al., 2019, 2021; Kharzinova, Zinovieva, 2020; Nosova et al., 2020; Lemesh et al., 2021).

To assess the condition and preserve the features of the KMM gene pool, genogeographic studies are needed. The preservation and further improvement of the breed should be controlled by the genetic dynamics studies both in the breed as a whole and in the main breeding farms engaged in KMM breeding. We have previously shown that local breeds of farm animals (in particular, the Kyrgyz horse) are characterized with a high genetic diversity, but local differentiation is also present, and the differences are significant for a number of high-altitude experimental zones (Isakova et al., 2021). In this regard, studies of similar structure are needed.

The information obtained during the molecular genetic analysis will complement the morphometric characteristics of breeding rams, repair rams and ewes, which will allow breeders to develop new and modify existing selection algorithms and schemes to maintain the inbreed KMM genetic diversity, as well as preserve the genetic identity of this breed. In the future, they plan a number of measures to improve the breeding qualities of KMM breed sheep.

Thus, the purpose of this study was to conduct a genogeographic study of the Kyrgyz mountain merino sheep breed.

## Materials and methods

**Fig. 1.**
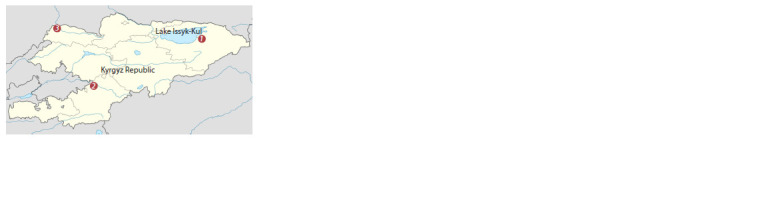
The sampling sites: 1 – SBF “Orgochor” (village Orgochor, Jety- Oguz district, Issyk-Kul region); 2 – SBF “Katta-Taldyk” (village Bash-Bulak, Karasu district, Osh region); 3 – SBF named after M.N. Lushchikhin (village of Dzhun-Tube, Kara-Burinsky district, Talas region).

The biological material for molecular genetic research was the blood samples of Kyrgyz mountain merino (KMM) sheep obtained from an adult population of 109 animals bred in three state breeding plants (SBF), including 29 animals from SBF “Orgochor” (village Orgochor, Jety-Oguz district, Issyk-Kul region) (ISSYK-KUL sample), 35 animals from SBF “Katta-Taldyk” (village Bash-Bulak, Karasu district, Osh region) (OSH sample) and 45 animals from SBF named after M.N. Lushchikhin (village of Dzhun-Tube, Kara-Burinsky district, Talas region) (TALAS sample). The sampling sites are shown in Fig. 1.

DNA was isolated by phenol-chloroform extraction (Sambrook, Russel, 2001). The samples were genotyped using 12 microsatellite markers recommended by the International Society for Animal Genetics (ISAG): McM042, INRA006, McM527, ETH152, CSRD247, OarFCB20, INRA172, INRA063, MAF065, MAF214, INRA005, INRA023, and also by the AMEL sex-specific locus.

Genotyping was carried out using a set of COrDIS Sheep (LLC “GORDIZ”, Russia) reagents for multiplex analysis according to the manufacturer’s recommendations. To correctly determine the genotype in the studied animals (amplicon size in bp), a sample with a control genotype included in the COrDIS Sheep kit was used. PCR were analyzed by capillary high-resolution electrophoresis using an automatic genetic analyzer Applied Biosystems 3500 (ThermoFisher, USA).

GenAIEx v. 6.503 (Peakall, Smouse, 2012), STRUCTURE v. 2.3.4 (Pritchard et al., 2000), Past v. 4.03 (Hammer et al., 2001) software was used for statistical analysis.

GenAIEx v. 6.503 was used to calculated the average number of alleles per locus (Na), the effective number of alleles (Ne), the levels of expected (He) and observed (Ho) heterozygosity and the FIS coefficient (Excoffier, 1991). STRUCTURE v. 2.3.4 allowed to calculate the Q criterion, which attributed each individual animal to the corresponding cluster (Pritchard et al., 2000). PPHELPER v. 1.0.10 web application (Francis, 2016) was used for graphical interpretation of the results obtained in STRUCTURE v. 2.3.4.

We used GenAlEx 6.503 software (Peakall et al., 2012) to analyze population genetic parameters, the degree of genetic differentiation based on matrices of pairwise FST values, followed by visualization in Past v. 4.03 (Hammer et al., 2001).

The genetic structure of the studied samples of the KMM sheep breed was evaluated using principal component analysis (PCA) via clustering in STRUCTURE v. 2.3.4 (Pritchard et al., 2000) using a mixed model (the number of assumed K clusters from 3 to 10; the length of the burn-in period 50K; the Markov chain model Monte Carlo 5K). Ten iterations were completed for each K value. We also determined the optimal number of clusters (ΔK) in POPHELPER v. 1.0.10 web application, using the method proposed in (Evanno et al., 2005).

All applicable international, national and/or institutional principles for the care and use of animals have been observed

## Results and discussion

The modern KMM sheep breed demonstrated a high level of inbreeding genetic variability, when 126 alleles were identified in the 12 microsatellite markers studied. The number of alleles in each locus varied from 6 to 16 (mean 10.500 ± 0.957). Sixty-seven rare alleles (with a prevalence less than 5.0 %) were identified, 53.2 % of the total number of identified alleles. The greatest number of rare alleles was found for the STR markers CSRD247, INRA023, INRA005, INRA006, MAF214 and OarFCB20.

In order to analyze KMM inbreeding genetic subdivision bred in three geographically isolated zones, we computed Na, Ne, Ho, He, I values and the FIS coefficient, shown in Table 1.

**Table 1.**
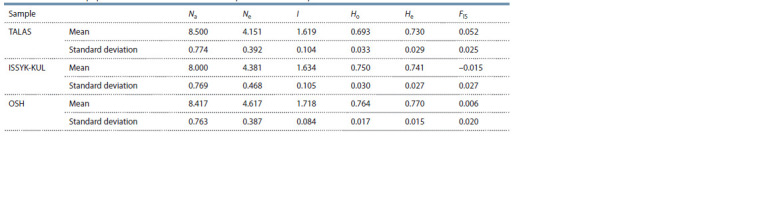
Genetic and population characteristics of three independent KMM samples based on 12 STR markers Notе. Na – No. of different alleles per locus; Ne – No. of effective alleles; I – Shannon’s information index; Ho – observed heterozygosity; He – expected heterozygosity; FIS – fixation index.

The mean number of alleles per Na locus varied from 8.000 to 8.500 (mean 8.306 ± 2.595), whereas the maximum value was noted in the TALAS group from the M.N. Lushchikhin SBF. The number of effective Ne alleles was the highest in the OSH sample from the Katta-Taldyk SBF. Shannon index, reflecting the complexity of the community structure, averaged 1.657 ± 0.333 with the highest value in the OSH sample from the Katta-Taldyk SBF. The observed heterozygosity Ho as an indicator of the variability (polymorphism) of the population reflecting the proportion of heterozygous genotypes in the experiment ranged from 0.693 to 0.764. The expected heterozygosity of He as an indicator of the proportion of heterozygous genotypes, expected in the Hardy–Weinberg equilibrium, ranged from 0.730 to 0.770. Maximum values of Ho and He were in OSH from the Katta-Taldyk SBF. The mean value of FIS index was the most neutral (0.006) in this group and indicated a balanced prevalence of heterogeneous genotypes, i. e. the level of related mating of individuals in the subpopulation was the least significant compared to the remaining two groups. In general, when comparing Na, Ne, Ho, He, I and the FIS coefficient, we found no statistically significant differences between three studied samples as of the Student’s t-test.

**Fig. 2.**
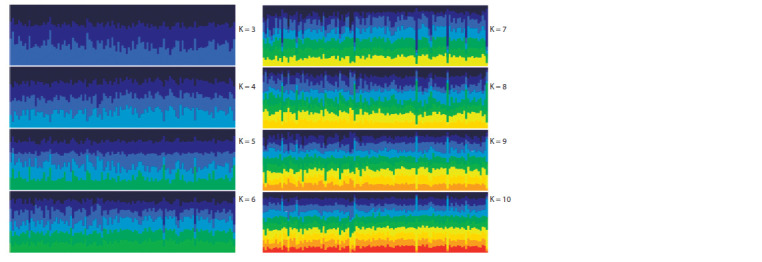
The analysis of the genetic structure of the studied KMM samples for the most probable number of clusters (K) from 3 to 10. X – axis is the ID of the animal; Y – axis is the proportion in the corresponding cluster; Q values are calculated using the method of (Pritchard et al., 2000).

To assess the genetic subdivision of the KMM samples using STRUCTURE v. 2.3.4, we computed the Q criterion, which characterized the stratification of each individual animal in the corresponding group. A Q value of 75 % or higher confirms the individual’s attribution to its cluster. Fig. 2 graphically demonstrates (using the PPHELPER v. 1.0.10 web application (http://pophelper.com/)) the results of the analysis carried out in STRUCTURE v. 2.3.4 (automatic sorting was carried out based on the attribution of a particular sample to a major cluster).

The genetic material of KMM sheep from three geographically isolated zones was used in the study (see Fig. 1). For all samples within clusters K = (3–10), there is a general uniformity of structure, whereas the contribution of each subcluster is equivalent. A pairwise comparison of the mean values of Q for three samples at K = 2 using analysis of variance showed no statistically significant differences. Thus, F = 0.112, p = 0.739 was for the pair TALAS/ISSYK-KUL; F = 0.023, p = 0.881, for the pair ISSYK-KUL/OSH; and F = 0.267, p = 0.607 was for the pair TALAS/OSH. This may result from the fact that the KMM subpopulations studied have common ancestors (for example, sheep producers); however, other factors may also have an effect.

**Fig. 3.**
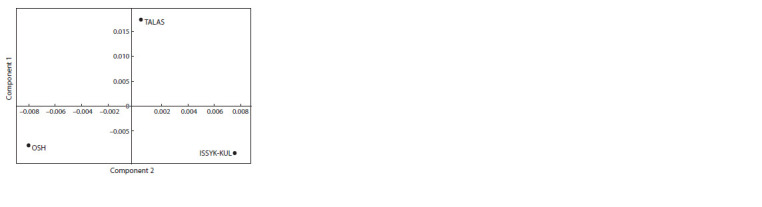
Results of the analysis of the main components (as of 12 STR-markers in total).

Based on the analysis of FST genetic distances calculated using the AMOVA algorithm for 12 STR markers, a PCR graph was constructed reflecting the mutual similarity/difference of the studied samples (Fig. 3)

The information presented in Fig. 2 and 3 allows to conclude that the studied samples of KMM did not differ significantly from each other. However, each sample had features that arose from the differences in the allele’s prevalence in the studied STR loci, as well as the presence of rare and private (found only in one of the studied groups) alleles (Tables 2 and 3, respectively).

**Table 2.**
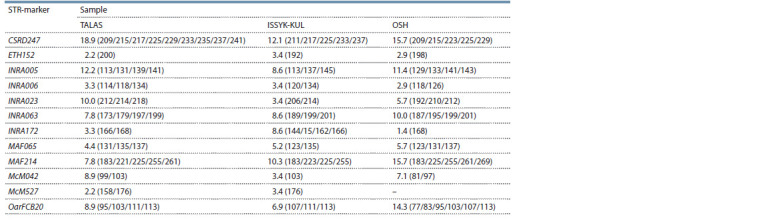
Total prevalence of rare alleles (prevalence less than 5 %) in the studied KMM samples in %

**Table 3.**
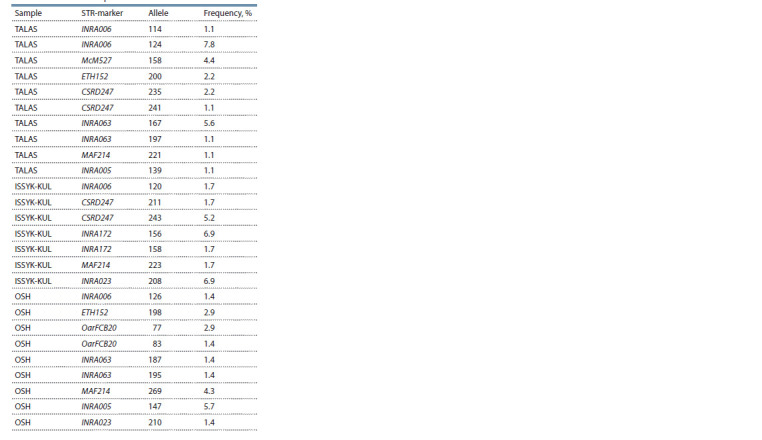
The prevalence of private alleles in the KMM studied samples

Among individuals from the M.N. Lushchikhin SBF (the TALAS sample), rare alleles accounted for 18.9, 12.2 and 10.0 %, respectively, for the CSRD247, INRA005 and INRA023 STR markers; among individuals from the Orgochor SBF (the ISSYK-KUL sample), high prevalence of rare alleles was found for STR markers CSRD247 and MAF214, 12.1 and 10.3 %, respectively; and among individuals from the Katta-Taldyk SBF (OSH sample) – for CSRD247 (15.7 %), MAF214 (15.7 %) and OarFCB20 (14.3 %).

In general, we found individual differences in the distribution profile of allele frequencies across all the studied STR loci for each group. The most significant of those were allele 87 in the major state in the McM042 locus (35.6 and 45.7 %, respectively) in the TALAS and OSH groups, whereas allele 95 was most prevalent (36.2 %) in the group ISSYK-KUL; major allele 154 for the INRA172 locus in all groups, however, in comparison with the TALAS group, its prevalence was 1.25 (ISSYK-KUL) and 1.66 (OSH) times lower, 55.2 and 41.4 %, respectively, and alleles 156 and 158 were found only in the ISSYK-KUL group; the prevalence of 186 allele in the ETH152 locus in the TALAS group was 51.1 %, whereas 190 allele was highly prevalent in ISSYK-KUL and OSH, 34.5 and 34.3 %, respectively.

We also identified specific peculiarities of private alleles. Among the sheep from the M.N. Lushchikhin SBF (TALAS) those were determined with regard to seven loci (a total of 10 alleles), including INRA006, McM527, ETH152, CSRD247, INRA063, MAF214 and INRA005; and for the INRA006 locus, the 124 allele was detected in 7.8 %. Seven private alleles in five STR markers were identified for sheep from the Orgochor SBF (ISSYK-KUL), the most common being INRA172 (allele 156, frequency – 6.9 %) and INRA023 (208, 6.9 %), as opposed to INRA005 (147, 5.7 %) for individuals from the Katta-Taldyk SBF (OSH).

The highest calculated FST values are shown for the loci McM042, INRA172 and ETH152, although in general the FST values for all loci were not high and did not exceed 0.05 ( p < 0.001).

We also conducted a comparative analysis of Na and He parameters for KMM and fine-wool sheep breeds bred in Kazakhstan (Dossybayev et al., 2019), Russia (Deniskova et al., 2016), Pakistan (Ahmed et al., 2014) and Poland (Szumiec et al., 2018) (Table 4).

**Table 4.**
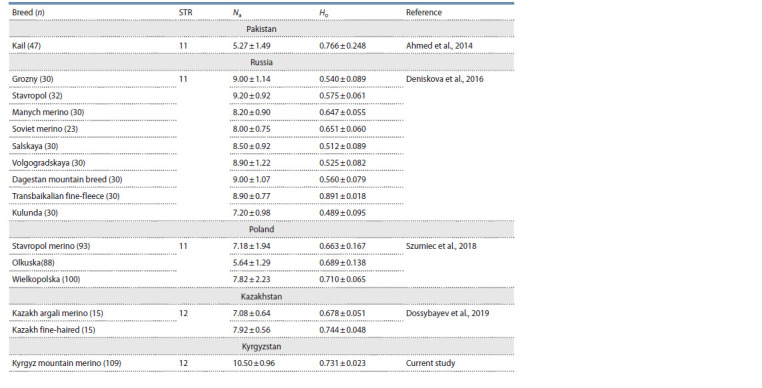
Genetic characteristics of fine-wool breeds sheep samples based on STR loci genotyping

We found that the mean Na in KMM (in the context of the STR markers studied in this paper) was the maximum in comparison with other studies. The calculated Ho index also turned out to be one of the largest and was comparable with the values obtained for the breeds Wielkopolskaya (Poland), Olkuska (Poland), Kail (Pakistan) and Kazakh fine-haired (Kazakhstan) (Ahmed et al., 2014; Szumiec et al., 2018; Dossybayev et al., 2019). The high rates of KMM genetic diversity are directly related to the multi-stage breeding processes that this breed underwent during the late XX– early XXI century.

## Conclusion

Taken together, the genetic diversity of KMM breed sheep of the three state breeding plants of the Kyrgyz Republic is quite high and comparable to each other. We found it impossible to single out a group for which a qualitatively different (high or low) genetic diversity would be different compared the other two groups.

Nevertheless, it cannot be denied that for Kyrgyz mountain merino sheep from the M.N. Lushchikhin SBF, there was still a slight shift towards inbreeding processes – FIS = = 0.052 ± 0.025 (the maximum individual values of this indicator were found for STR markers of INRA023 – 0.120, McM527 – 0.136, McM042 – 0.142 and MAF214 – 0.215). In this regard we assume that the positive shift of these markers (lack of heterozygotes) occurred due to the purposeful selection of individuals according to the economically valuable characteristics of wool, i. e. resulted from the association of these STR markers with the loci of quantitative traits QTL. However, such a relationship can only be assessed in further studies

An indirect confirmation of the inbreeding processes in this breeding plant may be the presence of six pairs of individuals among those selected for molecular genetic analysis, which were likely close relatives to each other (within pairs), because there were matching alleles in each of the 12 STR markers. In this regard, we propose to have a closer look at the intensity of inbreeding in the future. Four similar pairs were also identified among the individuals from the Orgochor and Katta-Taldyk SBFs, and it was possible that breeding events for the exchange of breeding sheep or repair sheep between these enterprises took place relatively recently.

## Conflict of interest

The authors declare no conflict of interest.
